# Palmitate reduces starvation-induced ER stress by inhibiting ER-phagy in hypothalamic cells

**DOI:** 10.1186/s13041-021-00777-8

**Published:** 2021-04-06

**Authors:** Yun Lim, Seolsong Kim, Eun-Kyoung Kim

**Affiliations:** 1 Department of Brain and Cognitive Sciences, Daegu Gyeongbuk Institute of Science and Technology (DGIST), Daegu, 42988 Republic of Korea; 2 Neurometabolomics Research Center, Daegu Gyeongbuk Institute of Science and Technology (DGIST), Daegu, 42988 Republic of Korea

**Keywords:** Palmitate, ER stress, Autophagy, ER-phagy, Starvation, Hypothalamic cells

## Abstract

Palmitate is a saturated fatty acid that is well known to induce endoplasmic reticulum (ER) stress and autophagy. A high-fat diet increases the palmitate level in the hypothalamus, the main region of the brain regulating energy metabolism. Interestingly, hypothalamic palmitate level is also increased under starvation, urging the study to distinguish the effects of elevated hypothalamic palmitate level under different nutrient conditions. Herein, we show that ER-phagy (ER-targeted selective autophagy) is required for progress of ER stress and that palmitate decreases ER stress by inhibiting ER-phagy in hypothalamic cells under starvation. Palmitate inhibited starvation-induced ER-phagy by increasing the level of B-cell lymphoma 2 (Bcl-2) protein, which inhibits autophagy initiation. These findings suggest that, unlike the induction of ER stress under nutrient-rich conditions, palmitate protects hypothalamic cells from starvation-induced stress by inhibiting ER-phagy.

**Supplementary Information:**

The online version contains supplementary material available at 10.1186/s13041-021-00777-8.

## Introduction

Consumption of high-fat diet (HFD) is one of the major factors leading to the development of obesity and related complications. HFD-induced obesity is characterized by increased circulating free fatty acids (FFAs), which are derived from adipocytes through lipolysis [1]. Specifically, the level of palmitate, a 16-carbon saturated fatty acid, is increased in the brain of both obese mice and humans [2, 3]. Since excessive palmitate induces lipotoxicity linked to obesity pathology [4], palmitate has been used in diet-induced obesity model at the cellular and organismal levels.

The hypothalamus is a brain region crucial for maintaining energy homeostasis by regulating food intake, energy expenditure, and the endocrine system in the development and progression of obesity in the context of excessive FFAs; HFD or administration of palmitate significantly induces hypothalamic inflammation [5, 6], resulting in the development of obesity. Studies using hypothalamic cell lines have revealed that palmitate directly modulates diverse cellular processes including insulin and leptin resistance, endoplasmic reticulum (ER) stress, and apoptosis [7–11], all of which affects hypothalamic functions.

Palmitate induces ER stress that activates the unfolded protein response (UPR) [12]. Stimuli such as aggregated proteins or unfolded proteins in the ER lumen provoke UPR to handle ER stress. There are three UPR sensors with distinct activation mechanisms: pancreatic ER kinase (PERK), inositol-requiring transmembrane kinase/endonuclease 1 (IRE1), and activating transcription factor 6 (ATF6). Phosphorylated PERK (p-PERK) activates activating transcription factor 4 (ATF4), and ATF4 activates CCAAT/enhancer-binding protein-homologous protein (CHOP). Phosphorylated IRE1 (p-IRE1) phosphorylates c-Jun N-terminal kinase, which activates CHOP. ER stress induces the transcription factor domain of ATF6 to enter the nucleus and regulate transcription of UPR target genes. The three sensors activate different pathways but also activate common adaptive processes for cell survival such as autophagy [13–15].

Macroautophagy (hereafter autophagy) is a lysosomal pathway for the degradation of aggregated proteins and damaged organelles to maintain cellular homeostasis [16]. Autophagy is usually considered as a non-selective process; however, the selective clearance of specific cellular organelles such as mitochondria, lipid droplets, and ER through autophagy, called selective autophagy, has been discovered [17–19]. Recently, ER-targeted autophagy named ER-phagy has been suggested to affect diverse physiological functions of ER such as protein synthesis and storage of calcium [20]. Four mammalian receptors for ER-phagy with different functions have been reported: FAM134B [19], RTN3 [21], SEC62 [22], and CCPG1 [23]. FAM134B and RTN3 participate in fragmentation of ER under starvation [19, 21]. CCPG1 takes part in ER stress-induced ER-phagy, whereas SEC62 mediates ER clearance only during the recovery from ER stress [22, 23].

Similar to HFD-induced obesity, fasting in a healthy state also increases the level of plasma FFAs [24], including palmitate, which arise from the hydrolysis of triglycerides in adipocytes [25]. During starvation, circulating FFAs are used as a primary fuel to maintain cellular energy status through fatty acid oxidation in most peripheral tissues including liver, muscle, and kidney [26–28], indicating the role of fasting FFAs as a nutritional source. Fasting-induced circulating FFAs reach the hypothalamus across the blood–brain barrier [29, 30]. They are rapidly taken up by hypothalamic neurons where they are initially esterified to triglycerides within lipid droplets and then broken down by autophagy, which in turn induces appetite-regulating neuropeptide expression [25, 31].

However, the role of FFAs in the hypothalamus during fasting, beyond regulation of neuropeptide expression, has not been well elucidated. Given the starvation-induced ER stress [32] and autophagy [31, 33–35] in hypothalamic cells, circulating palmitate under fasting might regulate ER stress and ER-phagy, affecting hypothalamic functions.

In this study, we sought to investigate the effect of palmitate on ER stress and ER-phagy in hypothalamic cells under starvation in comparison with nutrient-rich conditions. We demonstrated that palmitate increased ER stress and impaired ER-phagy by impeding autophagosome maturation under nutrient-rich conditions, but decreased ER stress and inhibited the initiation of ER-phagy under starvation. We discovered that ER-phagy is antecedent to ER stress and palmitate inhibits starvation-induced ER-phagy by increasing the expression of B-cell lymphoma 2 (Bcl-2). Thus, the blockade of ER-phagy by palmitate might alleviate the metabolic stress response under starvation. Taken together, these findings suggest not only a novel role of palmitate in ER stress and ER-phagy in response to starvation, but also its protective effect on nutrient deficiency-induced hypothalamic stress.

## Methods

### Antibodies and reagents

Antibodies were purchased from the following sources: CCPG1 (ab219854) and KDEL (ab12223) were from Abcam; RTN3 (PA2256) was from Boster Biological Technology; ATF4 (#11815), ATF6 (#65880), Bcl-2 (#2870), Beclin1 (#3738), CHOP (#5554), COX IV (#4844), GAPDH (#2118), IRE1α (#3294), p-PERK (Thr980, #3179), and PERK (#3192) were from Cell Signaling Technology; FAM134B (NBP2-55248) and p-IRE1α (Ser724, NB100-2323) were from Novus Biologicals; LC3 (L8918) and p62 (P0067) were from Sigma. The secondary antibodies were purchased from the following sources: Goat anti-rabbit IgG (H + L) secondary antibody (31460) was from Thermo Fisher Scientific and Alexa488-conjugated anti-mouse (715–545–150) was from Jackson ImmunoResearch. Reagents were purchased from the following source: Bafilomycin A1 (B1793), carbonyl cyanide 3-chlorophenylhydrazone (CCCP; C2759), and sodium phenylbutyrate (4PBA; SML0309) were from Sigma.

### Cell culture

N41 cells (mHypoE-N41, Cellutions Biosystems Inc., CLU121) were maintained in DMEM culture medium (Welgene, LM 001-07) supplemented with 10% fetal bovine serum (Corning, TCB101) and 1% penicillin/streptomycin (Hyclone Laboratories, SV30010) at 37 °C. Starvation was conducted by washing the cells twice with Earle’s buffered salt solution (EBSS; Welgene, LB002-01) and then incubating them in fresh EBSS for 3 h.

### Preparation of sodium palmitate

To prepare 100 mM palmitate solution, 27.8 mg of sodium palmitate (Sigma, P9767) was dissolved in 1 ml sterile water using a heating block at 70 °C for 10 min. Immediately after the incubation, 0.1 ml of 100 mM palmitate solution was added to 0.9 ml of serum-free DMEM containing 5% non-esterified fatty acid-free bovine serum albumin (Sigma, A9418), which was prepared at 40 °C, to produce 10 mM palmitate solution.

### siRNA and plasmid transfection

ON-TARGETplus siRNAs for scrambled control (D-001810–10-20), mouse *Bcl-2* (L-063933–00-0005), and mouse *Fam134b* (L-063361–01-0005) were purchased from Dharmacon. Cells were seeded in a 12-well plate for 24 h before transfection. Cells were transfected with siRNA (100 nM) using Lipofectamine 2000 (Invitrogen, 11,668–019) according to the manufacturer’s instructions. After 4 h, the medium was changed and the cells were incubated with fresh medium for 48 h.

For plasmid transfection, cells were transfected with 1 μg of plasmid using TurboFect Transfection Reagent (Thermo Fisher Scientific, R0534) according to the manufacturer’s instructions. The plasmids were as follows: pmRFP-GFP encoding tandem-fluorescent LC3 (mRFP-GFP-LC3; a gift from Dr. Inhee Mook-Jung, Seoul National University); pmRFP-LC3 (Addgene, #21075); pDsRed2-Mito (Clontech, #632421); pEGFP-DFCP1 (Addgene, #38269); pEGFP-Parkin was generated by subcloning Myc-Parkin from pRK5-Myc-Parkin (Addgene, #17612; Ted Dawson’s lab) into pEGFP-C1 (Clontech, #6084-1).

### Immunocytochemistry

Cells were grown on coverslips coated with poly-L-lysine (Sigma, P4707), fixed in 10% formalin for 10 min and permeabilized with 0.1% saponin (Sigma, 47036) in PBS for 15 min. After washing with PBS, the coverslips were incubated with primary antibody diluted with antibody diluent solution (Invitrogen, #00-3118) for 1 h at room temperature (RT) and washed with PBS. Then, the coverslips were incubated with secondary antibody for 2 h at RT. After washing with PBS, the nuclei were stained for 10 min with Hoechst 33,342 (Invitrogen, H3570) at a 1:3000 dilution in PBS. Finally, the coverslips were washed with PBS and mounted on microscope slides with ProLong Diamond Antifade Mountant (Thermo Fisher Scientific, P36970). Fluorescence images were examined under a confocal microscope (LSM 780 and LSM 800, Carl Zeiss) and analyzed in Zen software (Carl Zeiss).

### Western blotting

Cells were lysed in buffer containing 50 mM Tris–HCl, pH 7.4, 250 mM sucrose (Bioshop, SUC507), 5 mM sodium pyrophosphate (Sigma, S6422), 1 mM EDTA (Sigma, E5134), 1 mM EGTA (Sigma, E3889), 1% Triton X-100 (Sigma, T8787), 0.1 mM benzamidine (Sigma, B6506), 1 mM dithiothreitol (Sigma, #43816), 0.5 mM phenylmethylsulfonyl fluoride (Sigma, P7626), 50 mM sodium fluoride (Sigma, 201154), protease inhibitor cocktail (Calbiochem, #535140), and phosphatase inhibitor cocktail (Sigma, P5726). After 30 min of incubation on ice, lysates were centrifuged at 16,400 × *g* for 15 min and protein concentration in the supernatants was measured using a Pierce BCA protein assay kit (Thermo Fisher Scientific, #23225). Total protein (7 µg) was loaded on a SDS–polyacrylamide gel and blotted onto PVDF membranes (Millipore, IPVH00010) for 35 min at 20 V/cm in transfer buffer containing 25 mM Tris, 192 mM glycine, and 20% methanol. The membrane was blocked by 1 h incubation with 5% skim milk. After blocking, the membrane was incubated with the specific primary antibody for 1 h at RT or overnight at 4 °C. After three washes with 1 × TBST buffer containing 20 mM Tris, 125 mM NaCl, 0.1% Tween 20 (Sigma, P1379), pH 7.4, the membrane was incubated with secondary antibody and visualized by using ECL solutions (Thermo Fisher Scientific, #34580 and #34095) according to the recommended procedure. SRX 201A (Konica Minolta Medical Imaging) was used to develop films. Obtained images were analyzed using Image J software (National Institutes of Health) and the level of each protein was quantified and normalized to that of GAPDH.

### Real-time quantitative PCR

Total RNA from the N41 cells was extracted with TRIzol reagent (Invitrogen, #15596018), and its concentration was determined using a NanoDrop spectrophotometer (Thermo Scientific). RNA (3 µg) was reverse transcribed using a GoScript Reverse Transcription System (Promega, A5004) as described previously [34]. TB Green (TaKaRa Biotechnology, RR820A) was used for quantitative PCR estimation of the expression of *Beclin1* and *Gapdh* genes in a CFX 96 Real-Time system (Bio-Rad). The following primers were synthesized by Integrated DNA Technologies: *Beclin1* forward, 5ʹ-GGCGGCTCCTATTCCATC-3ʹ; *Beclin1* reverse, 5ʹ-TGAGGACACCCAAGCAAG-3ʹ; *Gapdh* forward, 5ʹ-ATCACTGCCACCCAGAAGAC-3ʹ; *Gapdh* reverse, 5ʹ-ACACATTGGGGGTAGGAACA-3ʹ. The expression level was normalized to that of *Gapdh* as an endogenous control. All assays were performed in triplicate.

### Statistical analysis

Data from at least three independent experiments were expressed as the mean ± standard error of the mean (SEM). Statistical significance was determined using one-way analysis of variance followed by Tukey’s multiple comparisons test using GraphPad Prism 8.0.1 (GraphPad Software).

## Results

### Palmitate induces ER stress and autophagy at early time points but prolonged palmitate treatment impairs autophagy under nutrient-rich conditions

**Fig. 1:**
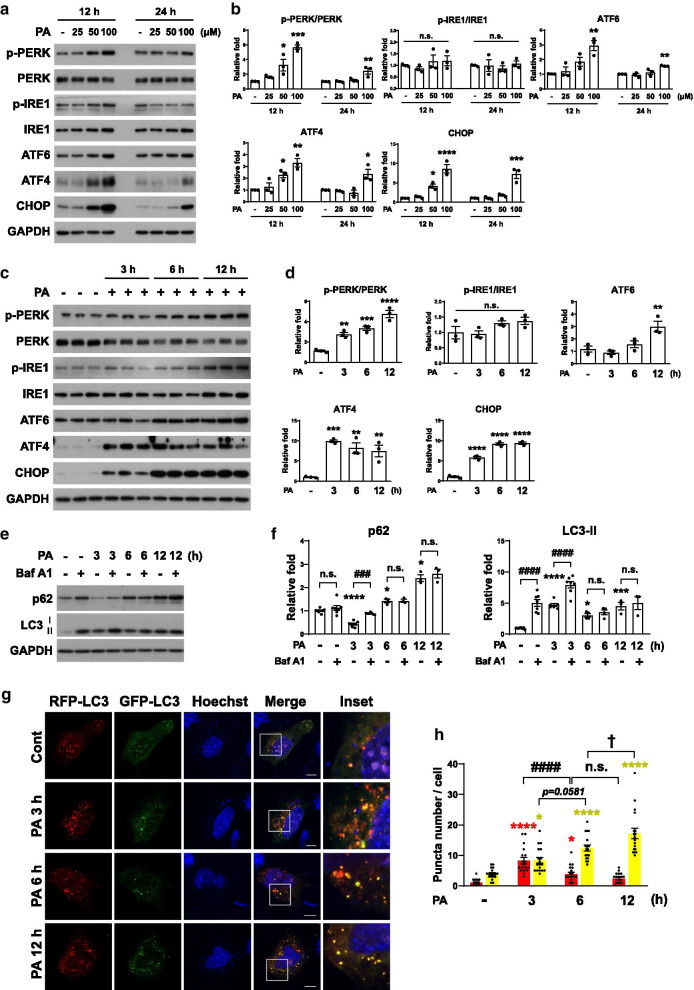
Palmitate induces ER stress and autophagy at early time points, but prolonged palmitate treatment impairs autophagy in a hypothalamic cell line. **a** and **b** Immunoblotting analysis (**a**) and quantification (**b**) of ER stress markers (p-PERK, PERK, p-IRE1, IRE1, ATF6, ATF4, and CHOP) in cells treated with different concentrations of palmitate (25, 50, and 100 μM) for the indicated times (n = 3 per each group). Data are mean ± SEM; p-PERK/PERK; *p = *0.0252*, ***p = *0.0003* vs. control at 12 h, **p = *0.*0065 vs. control at 24 h, ATF6; **p = *0.0040* vs. control at 12 h, **p = *0.0074* vs. control at 24 h, ATF4; *p = *0.0454*, **p = *0.0017* vs. control at 12 h, *p = *0.00139* vs. control at 24 h, CHOP; *p = *0.0240*, ****p < *0.0001* vs. control at 12 h, ***p = *0.0002* vs. control at 24 h. **c** and **d** Immunoblotting analysis (**c**) and quantification (**d**) of ER stress markers in cells treated with 0.1 mM palmitate for the indicated times (n = 3 per each group). Data are mean ± SEM; p-PERK/PERK; **p = *0.0050*, ***p = *0.0007*, ****p < *0.0001* vs. control, ATF6; **p = *0.0091* vs. control, ATF4; **p = *0.0033* at PA 6 h vs. control, **p = *0.0067* at PA 12 h vs. control, ***p = *0.0009* vs. control, CHOP; ****p < *0.0001* vs. control. **e** and **f** Immunoblotting analysis (**e**) and quantification (**f**) of autophagy markers (p62 and LC3-II) in cells treated with 0.1 mM palmitate for the indicated times. Baf A1 (200 nM) was added for 3 h before harvest (p62; n = 7 for control, control + Baf A1, PA 3 h, PA 3 h + Baf A1, n = 3 for PA 6 h, PA 6 h + Baf A1, PA 12 h, PA 12 h + Baf A1, LC3-II; n = 8 for control, n = 7 for control + Baf A1, PA 3 h, PA 3 h + Baf A1, n = 4 for PA 6 h, PA 6 h + Baf A1, n = 3 for PA 12 h, PA 12 h + Baf A1. Data are mean ± SEM; p62; *p = *0.0295* at PA 6 h vs. control, *p = *0.0250* at 12 h vs. control, ****p < *0.0001* vs. control, ^###^*p* = *0.0008*, LC3-II; *p = *0.0444*, ***p = *0.0003*, ****p < *0.0001* vs. control, ^####^*p* < *0.0001*. **g** and **h** Cells transiently expressing mRFP-GFP-LC3 were treated with 0.1 mM palmitate for the indicated times. Representative micrographs (**g**) and quantification (**h**) of the average number of mRFP-GFP-LC3 puncta per cell (n = 18 per each group). Scale bar, 10 μm. Data are mean ± SEM; Red puncta; *p = *0.0158*, ****p < *0.0001* vs. control, ^####^*p* < *0.0001*, Yellow puncta; *p = *0.0257*, ****p < *0.0001* vs. control, ^†^*p* = *0.0174*. n.s., no significant difference

To examine the effect of palmitate on ER stress in the mouse hypothalamic cell line N41 under normal nutrient-rich conditions, we checked the activation of three UPR sensor proteins, IRE1, PERK, and ATF6, following treatment with various concentrations of palmitate (Fig. 1a, b). Palmitate treatment for 12 h increased the levels of both p-PERK (50 and 100 μM) and ATF6 (100 μM), while p-IRE1 did not change (Fig. 1a, b). Also, the levels of ATF4 and CHOP, the late ER stress markers [36], were increased after 12 h treatment with palmitate (50 and 100 μM). The increases in the levels of p-PERK, ATF6, ATF4, and CHOP by palmitate at 24 h were less robust compared with 12 h treatment. Since the levels of ER stress markers were highest at 12 h with 100 μM of palmitate, the effect of this concentration was tested at three different early time points (Fig. 1c, d). While PERK was gradually phosphorylated, the level of ATF6 increased only at 12 h and the level of p-IRE1 did not change (Fig. 1c, d). A significant elevation in the levels of ATF4 and CHOP (Fig. 1c, d) suggests that the p-PERK–ATF4–CHOP axis is the major ER stress pathway activated by palmitate in N41 cells. Since ER stress regulates autophagy [37], we next assessed the effect of palmitate on autophagy in N41 cells. We used the levels of microtubule-associated protein 1 light chain 3 (LC3) and p62 as readouts for autophagy induction. When autophagy is induced, the LC3-I form is converted to LC3-II through lipidation [38]. At the same time, p62, a receptor protein that brings the cargo to the phagophore, is degraded [39]. To further monitor the autophagy flux induced by palmitate, we used bafilomycin A1 (Baf A1), a pharmacological inhibitor of autophagy [40]. Baf A1 blocks the fusion of autophagosomes with lysosomes, impairing autophagy flux and cargo degradation [40]. Therefore, the more accumulation of p62 and LC3-II after Baf A1 treatment indicates accumulated autophagosomes due to increased autophagy flux. Baf A1 co-treatment increased the levels of both p62 and LC3-II in comparison with palmitate alone only at 3 h but not at 6 or 12 h, suggesting that palmitate induced autophagy flux at 3 h but impaired autophagy at 6 and 12 h (Fig. 1e, f). In the mRFP-GFP-LC3 fluorescent puncta assay, since the RFP signal is more stable than GFP in acidic conditions, autophagy flux can be estimated by counting yellow puncta (autophagosomes) and red puncta (autolysosomes) [41]. Palmitate increased the total number of red plus yellow puncta at 6 and 12 h. However, based on a significantly higher ratio of yellow puncta over red, the increases in total puncta at 6 and 12 h are not due to increased autophagy influx but rather, to the accumulation of autophagosomes of which degradation is impaired (Fig. 1g, h). These data suggest that prolonged treatments with palmitate such as 6 and 12 h impaired autophagy by undermining autophagosome–lysosome fusion and autophagosome degradation. Also, considering that there are no increases in p62 and LC3-II at 6 and 12 h after Baf A1 treatment with palmitate, palmitate impaired autophagy at 6 and 12 h. Collectively, these results show that palmitate induces ER stress and autophagy at 3 h but impairs autophagy at 6 and 12 h.

### Palmitate impairs ER-phagy at the autophagosome maturation step

**Fig. 2:**
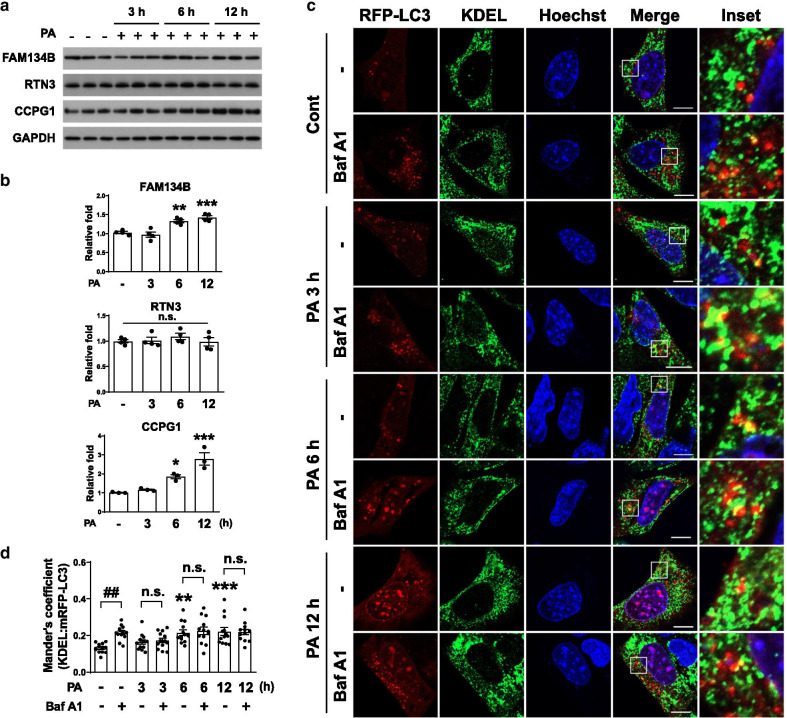
Palmitate impairs ER-phagy. **a** and **b** Immunoblotting analysis (**a**) and quantification (**b**) of ER-phagy receptors (FAM134B, RTN3, and CCPG1) in cells treated with 0.1 mM palmitate for the indicated times (FAM134B and RTN3; n = 4 per each group, CCPG1; n = 3 per each group). Data are mean ± SEM; FAM134B; **p = *0.0053*, ***p = *0.0005* vs. control, CCPG1; *p = *0.0388*, ***p = *0.0004* vs. control. **c** and **d** Cells transiently expressing mRFP-LC3 were treated with 0.1 mM palmitate for the indicated times in the presence or absence of Baf A1 (200 nM). Treated cells were stained for the ER marker KDEL. Representative micrographs (**c**) and quantification (**d**) of colocalization between mRFP-LC3 and KDEL (n = 13 per each group). Scale bar, 10 μm. Data are mean ± SEM; **p = *0.0029*, ***p = *0.0008* vs. control, ^##^*p* = *0.0025*. n.s., no significant difference

ER stress induced by the accumulation of unfolded proteins may drive ER-phagy to maintain ER proteostasis [23]. To examine whether palmitate-induced ER stress triggers ER-phagy in N41 cells, we measured the proteolytic degradation of ER-phagy receptors FAM134B, RTN3, and CCPG1 as the markers of ER-phagy [19, 21, 23] after palmitate treatment (Fig. 2a, b). Palmitate treatment for 6 or 12 h increased the levels of FAM134B and CCPG1 but had no effect on the RTN3 level (Fig. 2a, b). Because autophagy was impaired after 6 and 12 h palmitate treatment (Fig. 1e–h), the elevated FAM134B and CCPG1 levels suggested the impairment of ER-phagy. To visualize ER-phagy and distinguish ER-containing autophagosomes and autolysosomes in palmitate-treated cells, we analyzed the colocalization of mRFP-LC3 and KDEL (ER marker) in the presence or absence of Baf A1 (Fig. 2c). Colocalization was increased by Baf A1 in the absence of palmitate, showing ER-phagy at basal state (Fig. 2c, d). However, despite the overall increase in colocalization at 6 and 12 h, its level was not affected by Baf A1, indicating stalled ER-phagy at autophagosomes at these time points (Fig. 2c, d).

Palmitate might also regulate mitophagy, selective autophagy that degrades damaged mitochondria [42, 43]. To investigate whether palmitate induces mitophagy, we monitored selective translocation of Parkin (E3 ubiquitin ligase) from the cytosol to dysfunctional mitochondria, the well-known pathway of mitophagy [44]. The recruitment of Parkin to mitochondria was visualized by exogenous expression of GFP-Parkin and DsRed2-Mito (Additional file 1: Fig. S1a). While the treatment with the mitophagy-inducing protonophore CCCP showed clear recruitment of Parkin to mitochondria, there was no mitochondrial translocation of Parkin in palmitate-treated cells at any time point (Additional file 1: Fig. S1a, b). Consistently, the protein level of cytochrome *c* oxidase subunit 4 isoform 1 (COX IV), one of the mitochondrial marker proteins, was not changed by palmitate, but was significantly decreased by CCCP treatment (Additional file 1: Fig. S1c, d). Taken together, these results indicate that palmitate selectively impairs ER-phagy and does not affect mitophagy in N41 cells under nutrient-rich conditions.

### Short-term treatment with palmitate inhibits ER-phagy under starvation

**Fig. 3:**
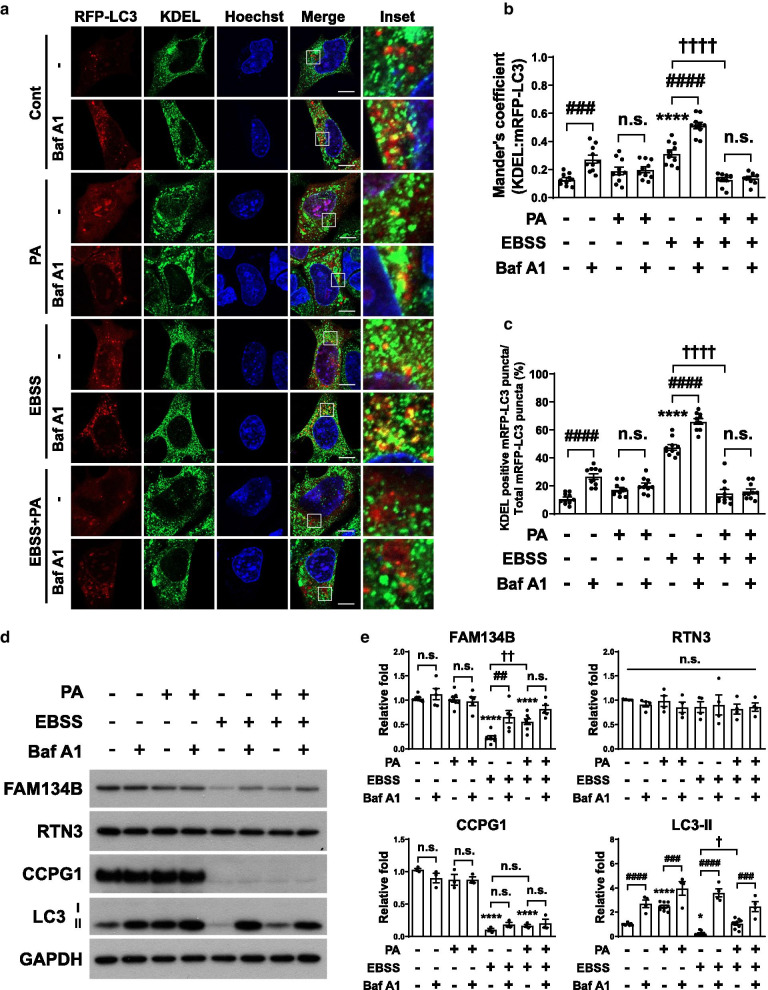
Palmitate inhibits EBSS-induced ER-phagy. **a**–**c** Cells transiently expressing mRFP-LC3 were starved in EBSS for 3 h in the presence or absence of palmitate (0.1 mM) and were co-treated for 3 h with Baf A1. Treated cells were stained for the ER marker KDEL. Representative micrographs (**a**) and quantification (**b**) of colocalization between mRFP-LC3 and KDEL (n = 10 per each group). Scale bar, 10 μm. Data are mean ± SEM; ****p < *0.0001* vs. control, ^###^*p* = *0.0002*, ^####^*p* < *0.0001*, ^††††^*p* < *0.0001*. **c** The ratio of the average number of KDEL-positive mRFP-LC3 puncta to the total number of mRFP-LC3 puncta per cell (n = 10 per each group). Data are mean ± SEM; ****p < *0.0001* vs. control, ^####^*p* < *0.0001*, ^††††^*p* < *0.0001*. **d** and **e** Immunoblotting analysis (**d**) and quantification (**e**) of ER-phagy receptors and LC3-II in cells starved in EBSS for 3 h in the presence or absence of palmitate (0.1 mM) and were co-treated for 3 h with Baf A1 (FAM134B; n = 8 for control, PA, EBSS, PA + EBSS, n = 5 for control + Baf A1, PA + Baf A1, EBSS + Baf A1, PA + EBSS + Baf A1, RTN3; n = 4 per each group, CCPG1; n = 3 per each group, LC3-II; n = 9 for control, PA, EBSS, PA + EBSS, n = 4 for control + Baf A1, PA + Baf A1, EBSS + Baf A1, PA + EBSS + Baf A1). Data are mean ± SEM; FAM134B; ****p < *0.0001* vs. control, ^##^*p* = *0.0016*, ^††^*p* = *0.0094*, CCPG1; ****p < *0.0001* vs. control, LC3-II; *p = *0.0331*, ****p < *0.0001* vs. control, ^###^*p* = *0.0002* at PA vs. PA + Baf A1, ^###^*p* = *0.0005* at PA + EBSS vs. PA + EBSS + Baf A1, ^####^*p* < *0.0001*, ^†^*p* = *0.0203*. n.s., no significant difference

Since ER-phagy was impaired by prolonged palmitate treatment (≥ 6 h) under nutrient-rich conditions, we further examined whether palmitate also inhibits starvation-induced ER-phagy. ER-phagy is highly upregulated in response to nutrient deprivation such as nitrogen starvation [45] and EBSS [23]. The incubation of hypothalamic cells in EBSS for 3 h significantly increased the colocalization of mRFP-LC3 and KDEL, and the treatment with Baf A1 further increased the colocalization due to the blockage of KDEL-containing proteins degradation and their accumulation in autophagosomes (Fig. 3a, b). Interestingly, the treatment of starved cells with palmitate for 3 h, the time point at which palmitate did not impair autophagy under nutrient-rich conditions, greatly reduced the colocalization (Fig. 3a, b). Since Baf A1 had no additional effect on this reduction, palmitate might block an early stage of the autophagic process such as autophagosome formation, rather than degradation of ER compartments. To determine the difference in the proportion of ER-phagy in the overall autophagic process induced by EBSS and/or palmitate, we analyzed the ratio of the number of KDEL-positive mRFP-LC3 puncta to total mRFP-LC3 puncta (Fig. 3c). Whereas the ratio of ER-phagy after palmitate treatment (17%) was similar to that in basal state (11%) or additional Baf A1 treatment (20%), EBSS-induced ER-phagy accounted for about 50% of total autophagy and its ratio was dramatically decreased by palmitate (15%) similar to the basal ER-phagy ratio, implying that palmitate almost completely inhibits ER-phagy induced by EBSS.

Next, we assessed which ER-phagy cargo receptors are involved in the inhibition of ER-phagy by palmitate under starvation (Fig. 3d, e). EBSS significantly decreased the levels of both FAM134B and CCPG1, whereas additional treatment with palmitate or Baf 1A increased the level of FAM134B only. The level of LC3-ll was also decreased by EBSS due to rapid degradation, evident from the accumulation of the protein in cells treated with palmitate or Baf A1 under starvation. While treatment of Baf A1 with EBSS significantly increased FAM134B compared to EBSS only, the treatment of Baf A1 with palmitate plus EBSS did not change FAM134B compared to palmitate plus EBSS, suggesting that palmitate inhibits the degradation of FAM134B under starvation. However, the level of RTN3 was not changed by EBSS, palmitate, or Baf A1. Taken together, these data suggest that, unlike under nutrient-rich conditions, palmitate treatment for 3 h inhibits EBSS-induced ER-phagy, which is mediated, at least in part, by FAM134B.

### ER-phagy precedes ER stress and palmitate reduces ER stress by inhibiting ER-phagy under starvation

**Fig. 4:**
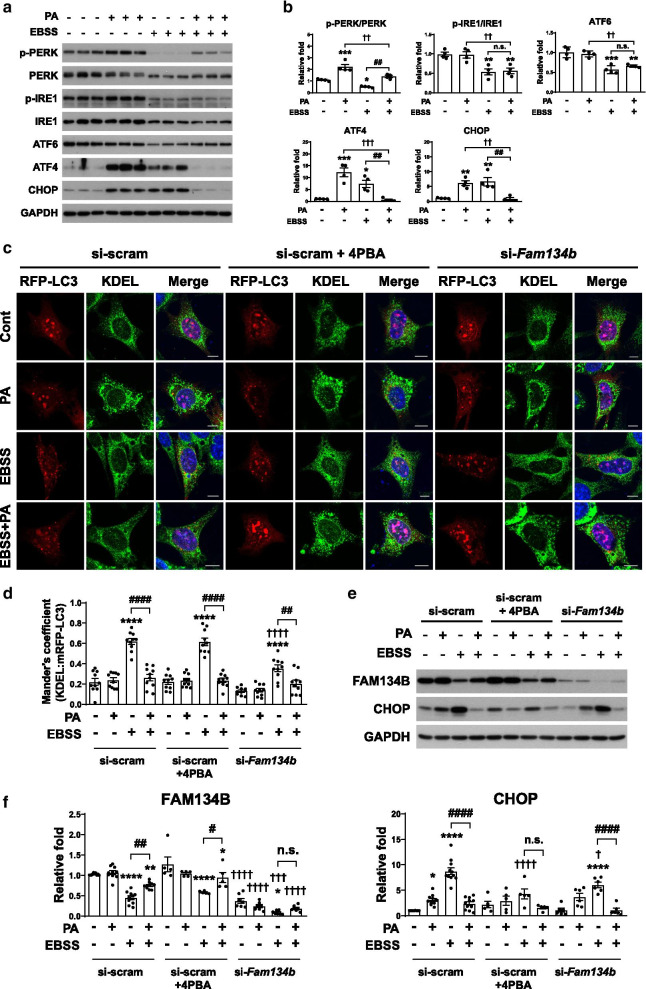
Inhibitory effect of palmitate on EBSS-induced ER-phagy precedes EBSS-induced ER stress. **a** and **b** Immunoblotting analysis (**a**) and quantification (**b**) of ER stress markers in cells starved in EBSS for 3 h in the presence or absence of palmitate (n = 4 per each group). Data are mean ± SEM; p-PERK/PERK; *p = *0.0220*, ***p = *0.0002* vs. control, ^##^*p* = *0.0012*, ^††^*p* = *0.0021*, p-IRE1/IRE; **p = *0.0038* at EBSS vs. control, **p = *0.0060* at PA + EBSS vs. control, ^††^*p* = *0.0072*, ATF6; **p = *0.0012*, ***p = *0.0001* vs. control, ^††^*p* = *0.0032*, ATF4; *p = *0.0146*, ***p = *0.0002* vs. control, ^##^*p* = *0.0094*, ^†††^*p* = *0.0001*, CHOP; **p = *0.0045* at PA vs. control, **p = *0.0022* at EBSS vs. control, ^##^*p* = *0.0016*, ^††^*p* = *0.0032*. **c**–**f** Cells transfected with mRFP-LC3 24 h after transfection with si-scram or si-*Fam134b* were starved in EBSS for 3 h in the presence or absence of palmitate (0.1 mM). Cells transfected with si-scram were pretreated with 4PBA (5 mM) for 1 h before starvation. After all treatments, cells were stained for the ER marker KDEL. **c** and **d** Representative micrographs (**c**) and quantification (**d**) of colocalization between mRFP-LC3 and KDEL (n = 10 per each group). Scale bar, 10 μm. Data are mean ± SEM; ****p < *0.0001* vs. each control in si-scram, si-scram + 4PBA, si-*Fam134b*, ^##^*p* = *0.0097*, ^####^*p* < *0.0001*, ^††††^*p* < *0.0001* vs. EBSS in si-scram. **e** and **f** Immunoblotting analysis (**e**) and quantification (**f**) of FAM134B and CHOP (FAM134B; n = 10 for si-scram, n = 5 for si-scram + 4PBA, n = 7 for si-*Fam134b* group, CHOP; n = 11 for si-scram, n = 5 for si-scram + 4PBA, n = 6 for si-*Fam134b* group). Data are mean ± SEM; FAM134B; **p = *0.0051* at PA + EBSS in si-scram vs. control in si-scram, ****p < *0.0001* at EBSS in si-scram vs. control in si-scram, *p = *0.0419* at PA + EBSS in si-scram + 4PBA vs. control in si-scram + 4PBA, ****p < *0.0001* at EBSS in si-scram + 4PBA vs. control in si-scram + 4PBA, *p = *0.0295* at EBSS in si-*Fam134b* vs. control in si-*Fam134b*, ^#^*p* = *0.0139*, ^##^*p* = *0.0013*, ^†††^*p* = *0.0004* at EBSS in si-*Fam134b* vs. EBSS in si-scram, ^††††^*p* < *0.0001* vs. each control, PA, PA + EBSS in si-scram, CHOP; *p = *0.0372* vs. control in si-scram, ****p < *0.0001* at EBSS in si-scram vs. control in si-scram, ****p < *0.0001* at EBSS in si-*Fam134b* vs. control in si-*Fam134b*, ^####^*p* < *0.0001*, ^†^*p* = *0.0170* vs. EBSS in si-scram, ^††††^*p* < *0.0001* vs. EBSS in si-scram. n.s., no significant difference

Next, we examined whether palmitate regulates starvation-induced ER stress. Starvation increased the levels of ATF4 and CHOP, but decreased those of the upstream UPR sensor proteins such as p-PERK, p-IRE1, and ATF6 (Fig. 4a, b), suggesting that EBSS induces ER stress independently of the three UPR sensors [32]. Interestingly, the levels of ATF4 and CHOP increased by EBSS were greatly diminished by palmitate (Fig. 4a, b). Thus, these results demonstrate that palmitate reduces starvation-induced ER stress.

To investigate the relationship between ER-phagy and ER stress under starvation, both of which are inhibited by palmitate, we inhibited ER stress and ER-phagy by 4PBA pretreatment and by knockdown of *Fam134b*, respectively, and then examined the markers of ER-phagy and ER stress in cells treated with EBSS and/or palmitate (Fig. 4c–f). The colocalization of mRFP-LC3 and KDEL was significantly reduced by *Fam134b* knockdown under EBSS conditions, but no change was observed in 4PBA pretreatment, suggesting that ER-phagy was not affected by ER stress under starvation (Fig. 4c, d). The inhibition of the colocalization of ER and autophagosomes by palmitate was maintained in both 4PBA pretreatment and *Fam134b* knockdown under starvation (Fig. 4c, d), consistent with palmitate-induced accumulation of FAM134B in 4PBA pretreatment under starvation (Fig. 4e, f). However, *Fam134b* knockdown did not lead to any accumulation of FAM134B induced by palmitate under starvation (Fig. 4e, f). Thus, these results indicate that palmitate inhibits ER-phagy in an ER stress- and *Fam134b*-independent manner. On the contrary, the level of CHOP was significantly decreased by *Fam134b* knockdown under starvation, demonstrating that ER-phagy precedes and is required for ER stress under starvation. Decreased level of CHOP by palmitate under EBSS in *Fam134b*-knockdown cells compared to that of EBSS conditions in *Fam134b*-knockdown cells might be due to sustained inhibition of ER-phagy by palmitate (Fig. 4e, f). Therefore, palmitate reduces ER stress by inhibiting ER-phagy under starvation.

### Palmitate inhibits ER-phagy at the initiation step by increasing Bcl-2 level under starvation

**Fig. 5:**
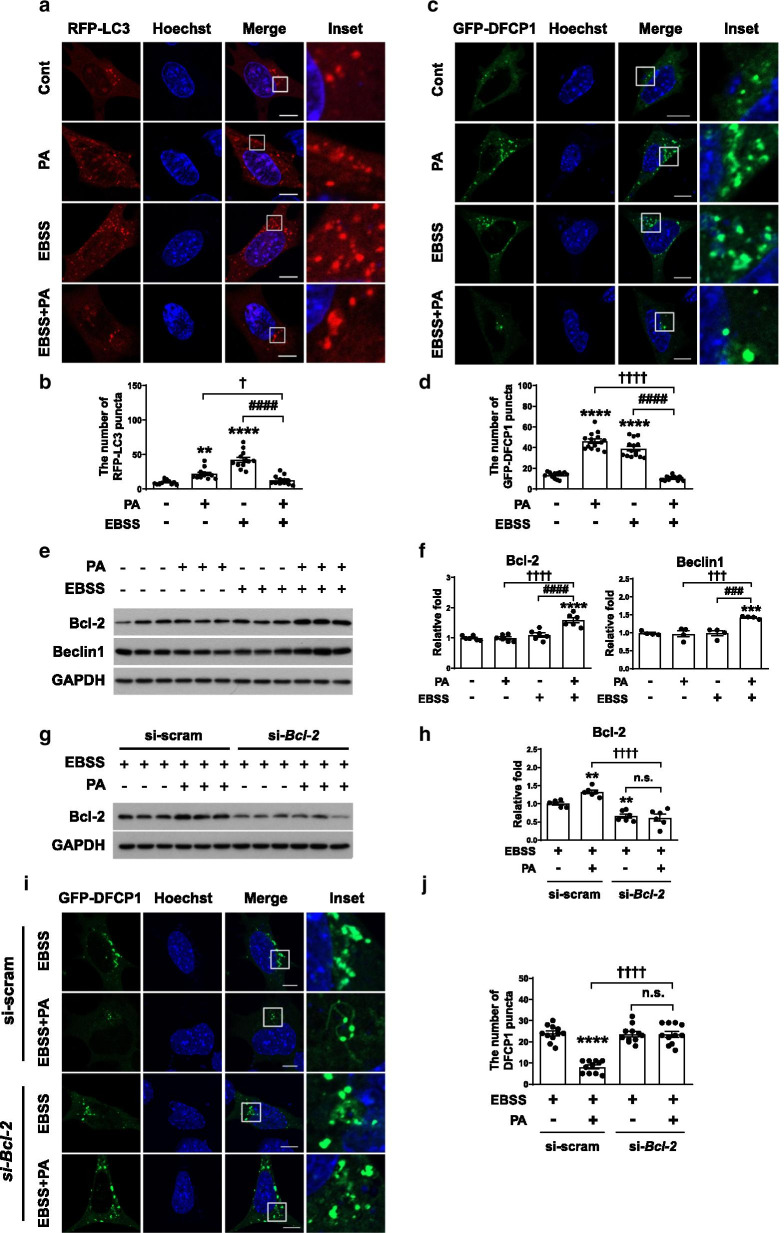
Palmitate inhibits ER-phagy via upregulation of Bcl-2. **a**–**f** Cells were starved in EBSS for 3 h in the presence or absence of palmitate (0.1 mM). **a** and **b** Representative micrographs (**a**) and quantification (**b**) of the average number of mRFP-LC3 puncta in cells transiently expressing mRFP-LC3 (n = 12 per each group). Scale bar, 10 μm. Data are mean ± SEM; **p = *0.0032*, ****p < *0.0001* vs. control, ^####^*p* < *0.0001*, ^†^*p* = *0.0358*. **c** and **d** Representative micrographs (**c**) and quantification (**d**) of the average number of GFP-DFCP1 puncta in cells transiently expressing GFP-DFCP1 (n = 14 per each group). Scale bar, 10 μm. Data are mean ± SEM; ****p < *0.0001* vs. control, ^####^*p* < *0.0001*, ^††††^*p* < *0.0001*. **e** and **f** Immunoblotting analysis (**e**) and quantification (**f**) of Bcl-2 and Beclin1 (Bcl-2; n = 6 per each group, Beclin1; n = 4 per each group). Data are mean ± SEM; Bcl-2; ****p < *0.0001* vs. control, ^####^*p* < *0.0001*, ^††††^*p* < *0.0001*, Beclin1; ***p = *0.0007* vs. control, ^###^*p* = *0.0007*, ^†††^*p* = *0.0005*. **g**–**j** Cells were transfected with si-scram or si-*Bcl-2* were starved in EBSS for 3 h in the presence or absence of palmitate (0.1 mM). **g** and **h** Immunoblotting analysis (**g**) and quantification (**h**) of Bcl-2 (n = 6 per each group). Data are mean ± SEM; **p = *0.0089* at EBSS + PA in si-scram vs. EBSS in si-scram, **p = *0.0054* at EBSS in si-*Bcl-2* vs. EBSS in si-scram, ^††††^*p* < *0.0001*. **i** and **j** Representative micrographs (**i**) and quantification (**j**) of the average number of GFP-DFCP1 puncta in cells transiently expressing GFP-DFCP1 (n = 11 per each group). Scale bar, 10 μm. Data are mean ± SEM; ****p < *0.0001* vs. EBSS in si-scram, ^††††^*p* < *0.0001*. n.s., no significant difference

Next, we investigated how palmitate prevents ER-phagy under starvation. We counted the number of mRFP-LC3 puncta, which represent autophagosomal compartments [46], in cells treated with EBSS and/or palmitate treatment (Fig. 5a, b). Each treatment increased the number of mRFP-LC3 puncta, whereas combined treatment decreased it to a level similar to that of control, suggesting that palmitate decreased autophagy under starvation. We also performed the experiments using double FYVE-domain-containing protein 1 (DFCP1), an early autophagy marker [47], to check the initiation of autophagosome formation (Fig. 5c, d). Similar to the result of mRFP-LC3 puncta, the number of GFP-DFCP1 puncta was decreased in co-treatment with palmitate and EBSS while that was increased by either palmitate or EBSS, confirming that palmitate decreased autophagosome formation under starvation. The autophagy-related (ATG) proteins are necessary for the biogenesis of autophagosomes. Although it was reported that palmitate inhibits autophagy by degrading ATG5 [48] or decreasing the expression of ATG7 [49], the protein levels of ATG5 and ATG7 were not changed by palmitate treatment under starvation (Additional file 2: Fig. S2a, b).

Bcl-2, an antiapoptotic protein, is known to inhibit Beclin1-dependent autophagy [50]. To examine whether palmitate-inhibited ER-phagy in starved cells is regulated by Bcl-2, we analyzed the Bcl-2 level. Interestingly, Bcl-2 and Beclin1 levels were not changed by either palmitate or EBSS, whereas co-treatment significantly increased the levels of both Bcl-2 and Beclin1 (Fig. 5e, f), suggesting that the increase in Bcl-2 might be involved in palmitate-inhibited ER-phagy. To confirm whether Bcl-2 contributes to inhibition of autophagy, Bcl-2 was knocked down under EBSS or co-treatment with palmitate and EBSS conditions (Fig. 5g, h). A decrease in the number of GFP-DFCP1 puncta under co-treatment with palmitate and EBSS conditions was reversed by the knockdown of *Bcl-2* in the same conditions (Fig. 5i, j), suggesting that Bcl-2 contributes to inhibition of autophagy initiation by palmitate under starvation. Taken together, these data suggest that ER-phagy is inhibited by palmitate at an early stage through the upregulation of Bcl-2 under starvation.

## Discussion

The effects of FFAs on ER stress, autophagy, and apoptosis in the brain have been investigated mainly in the context of mimicking excess-nutrient conditions such as HFD in vivo [51]. However, since FFA levels are increased in the hypothalamus not only by HFD but also under starvation, the role of FFAs in both contexts needs to be further explored. Palmitate, a saturated fatty acid, induces ER stress and apoptosis in hypothalamic cells [7–9], but the effect of palmitate on ER stress and apoptosis under starvation has not been studied. Given that starvation induces ER stress, which then upregulates ER-phagy [23], whether palmitate regulates ER stress and ER-phagy under starvation needs to be clarified. Here, we demonstrate a different role of palmitate in ER stress and ER-phagy under starvation in comparison with nutrient-rich conditions.

In our study, both palmitate and EBSS-induced starvation triggered ER stress through the ATF4-CHOP pathway, but through different upstream regulators. Under normal nutrient-rich conditions, palmitate induced ER stress through p-PERK signaling. On the other hand, EBSS degraded the three UPR sensors; therefore, starvation seems to induce ER stress in an UPR sensor–independent manner (Fig. 4a, b). Since both palmitate and EBSS increased ER stress, combined treatment could be expected to synergistically activate ER stress. However, opposite to this expectation, co-treatment with palmitate and EBSS greatly decreased the level of ER stress in comparison with that under each treatment, suggesting that palmitate may protect hypothalamic N41 cells against starvation-induced metabolic stress by reducing ER stress.

We also demonstrated that palmitate differently regulates ER-phagy depending on nutrient status. Under nutrient-rich conditions, our data showed the existence of basal ER-phagy, which was not affected by short-term treatment with palmitate, but was impaired by prolonged palmitate treatment. Starvation, on the other hand, significantly induced ER-phagy, which accounted for half of total autophagy. Surprisingly, short palmitate treatment inhibited starvation-induced ER-phagy close to basal level (Fig. 3a, b). Overall, palmitate inhibited both ER-phagy and ER stress under starvation (Figs. 3a, b and 4a, b). Furthermore, we showed that ER-phagy precedes ER stress and is required for ER stress under starvation (Fig. 4c–f). Although many studies have reported that ER stress induces autophagy, including ER-phagy [23, 52–54], the opposite is also possible because excessive ER-phagy may cause ER stress [55].

One interesting finding in our study is that palmitate yields opposite levels of autophagy flux depending on treatment duration under nutrient-rich conditions. Acute and chronic exposure of cells to palmitate can elicit Ca^2+^ influx through distinct entry mechanisms with different physiological outcomes [56]. Since autophagy can be induced or suppressed by various intracellular modulators, including Ca^2+^ [57–61], we speculate that short vs. long treatment of palmitate may have different effects on intracellular Ca^2+^ signaling, and subsequently autophagy.

One study demonstrated that long treatment with palmitate (16 h) impaired autophagy by suppressing the conversion from LC3-I to LC3-II and accumulating p62 level despite increasing the number and size of autophagosome in nutrient-rich conditions [62]. Although the study showed that palmitate overwhelmingly impaired autophagy even under amino acids starvation, the short-time effect of palmitate on autophagy under starvation or the type of autophagy was not investigated. Interestingly, our data showed that short treatment with palmitate (3 h) impaired autophagy, especially ER-phagy, by inhibiting the initiation of autophagosome formation under starvation.

We propose a mechanism by which palmitate inhibits autophagosome formation to inhibit ER-phagy under starvation (Fig. 5a–d). Under nutrient-rich conditions, palmitate inhibits autophagy by degrading ATG5 or ATG7 protein in several cell lines [48, 49], but the effect of palmitate on the regulation of ATG genes or proteins in hypothalamic cells has not been studied. Our results show that neither ATG5 nor ATG7 is affected by palmitate under nutrient-rich or starvation conditions in hypothalamic cells (Additional file 2: Fig. S2a, b), indicating that neither of these proteins participates in the inhibition of autophagy by palmitate.

Bcl-2 inhibits autophagy through the interaction with Beclin1 [50, 63]. Therefore, modulation of Bcl-2 level can be an effective mechanism to suppress or promote autophagy [64]. In our study, the levels of Bcl-2 and Beclin1 were not changed by either palmitate or EBSS-induced starvation, but were increased by co-treatment with EBSS and palmitate (Fig. 5e, f). Since the mRNA level of *Beclin1* was not changed by EBSS and/or palmitate (Additional file 3: Fig. S3) and the increased protein level of Beclin1 by co-treatment with EBSS and palmitate was diminished in *Bcl-2* knockdown cells (Additional file 4: Fig. S4a, b), the increase in Beclin1 protein by palmitate is likely due to Bcl-2-mediated Beclin1 stability at this conditions.

**Fig. 6:**
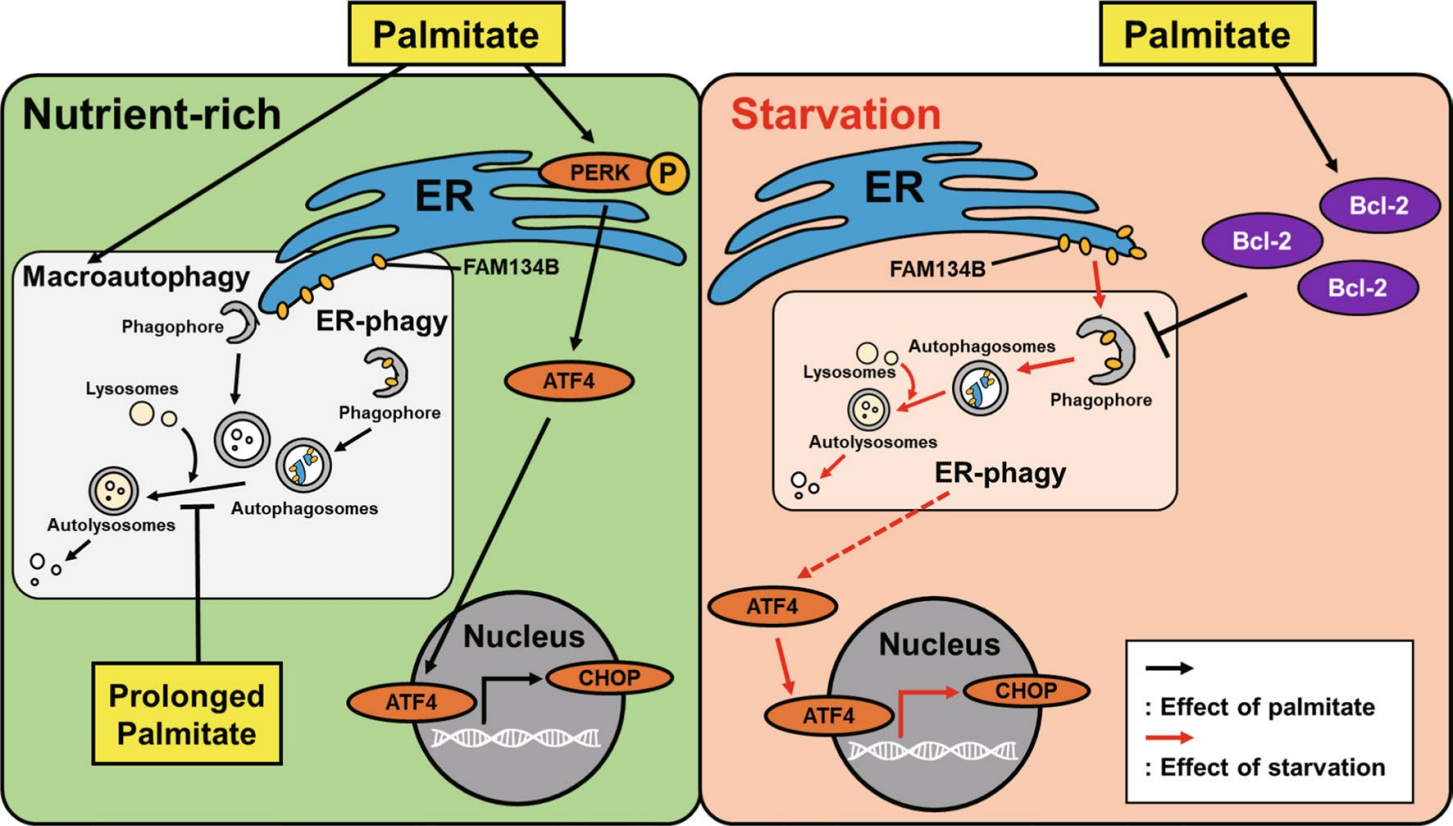
A schematic diagram illustrating the different roles of palmitate in ER-phagy and ER stress under nutrient-rich and starvation conditions. Palmitate induces p-PERK-mediated ER stress and autophagy, but prolonged treatment with palmitate impairs autophagy under nutrient-rich conditions. EBSS-induced starvation increases ER-phagy and ER stress, and ER-phagy precedes ER stress under these circumstances. Palmitate inhibits ER-phagy by increasing Bcl-2 and also decreases ER stress under EBSS-induced starvation

Taken together, our data demonstrate that palmitate differently regulates ER-phagy and ER stress depending on nutrient conditions (Fig. 6). Palmitate inhibits both ER-phagy and ER stress under starvation, relieving cellular stress. Further studies on the role of palmitate in the regulation of ER-phagy in the hypothalamus under fasting in vivo will be needed. In conclusion, our findings suggest that palmitate may work as a regulator of ER-phagy to protect cells under starvation.

## Supplementary Information

**Additional file 1: Fig. S1.** Palmitate does not induce mitophagy. **a**–**d** Cells were treated with 0.1 mM palmitate for the indicated times. CCCP (50 μM) was added for 3 h as the positive control for mitophagy. **a** and **b** Cells transiently expressing DsRed-Mito and GFP-Parkin were treated with 0.1 mM palmitate for the indicated times. Representative micrographs (**a**) and quantification (**b**) of colocalization between DsRed-Mito and GFP-Parkin (n = 11 per each group). Scale bar, 10 μm. Data are mean ± SEM; ****p < *0.0001* vs. control. **c** and **d** Immunoblotting analysis (**c**) and quantification (**d**) of the mitochondrial marker COX IV and LC3-II (n = 4 per each group). Data are mean ± SEM; COX IV; *p = *0.0291* vs. control, LC3-II; ****p < *0.0001* vs. control.

**Additional file 2: Fig. S2.** Palmitate does not change the levels of ATG5 and ATG7. Cells were starved in EBSS for 3 h in the presence or absence of palmitate (0.1 mM). **a** and **b** Immunoblotting analysis (**a**) and quantification (**b**) of ATG5 and ATG7 (n = 4 per each group). Data are mean ± SEM; n.s., no significant difference.

**Additional file 3: Fig. S3.** The mRNA level of *Beclin1* is not changed by palmitate and/or EBSS. Cells were starved in EBSS for 3 h in the presence or absence of palmitate (0.1 mM). **a** mRNA expression of *Beclin1* (n = 9 per each group). Data are mean ± SEM; ^†††^*p* = *0.0008*. n.s., no significant difference.

**Additional file 4: Fig. S4.** Knockdown of *Bcl-2* diminishes the increase of Beclin1 by palmitate under starvation. Cells transfected with si-scram or si-*Bcl-2* were starved in EBSS for 3 h in the presence or absence of palmitate (0.1 mM). **a** and** b** Immunoblotting analysis (**a**) and quantification (**b**) of Beclin1 (n = 6 per each group). Data are mean ± SEM; **p = *0.0052* vs. EBSS in si-scram, ^††^*p* = *0.0019*. n.s., no significant difference.

## Data Availability

All data generated or analyzed during this study are included in this published article.

## Abbreviations

ATF4: Activating transcription factor 4
ATF6: Activating transcription factor 6
ATG: Autophagy-related
Baf A1: Bafilomycin A1
Bcl-2: B-cell lymphoma 2
CCCP: Carbonyl cyanide 3-chlorophenylhydrazone
CHOP: CCAAT/enhancer-binding protein homologous protein
COX IV: Cytochrome c oxidase subunit 4 isoform 1
DFCP1: Double FYVE-domain-containing protein 1
EBSS: Earle’s buffered salt solution
ER: Endoplasmic reticulum
FFAs: Free fatty acids
GFP: Green fluorescent protein
HFD: High-fat diet
IRE1: Inositol-requiring transmembrane kinase/endonuclease 1
LC3: Microtubule-associated protein 1 light chain 3
PERK: Pancreatic ER kinase
RFP: Red fluorescent protein
UPR: Unfolded protein response
4PBA: Sodium phenylbutyrate
