# Supplementary figures and images for: Palmitate reduces starvation-induced ER stress by inhibiting ER-phagy in hypothalamic cells
Source: Mol Brain. 2021 Apr 6;14:65. doi: 10.1186/s13041-021-00777-8 (PMC8025501; doi:10.1186/s13041-021-00777-8)

Fig. S1

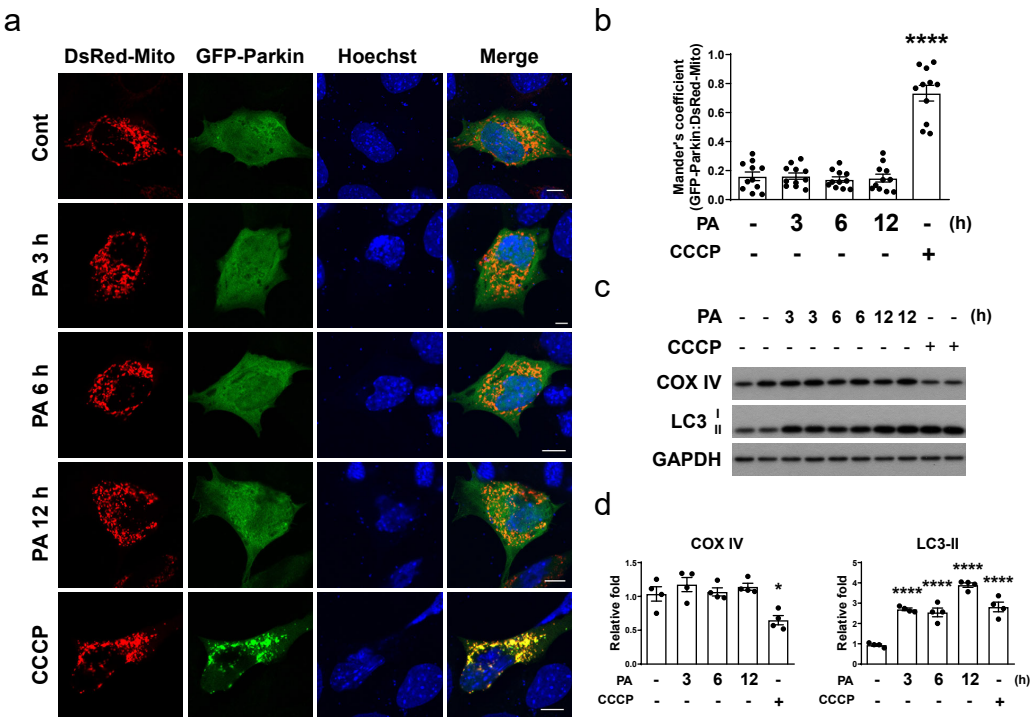

Supplement: Supplementary file 1 — Additional file 1: Fig. S1. Palmitate does not induce mitophagy. a–d Cells were treated with 0.1 mM palmitate for the indicated times. CCCP (50 μM) was added for 3 h as the positive control for mitophagy. a and b Cells transiently expressing DsRed-Mito and GFP-Parkin were treated with 0.1 mM palmitate for the indicated times. Representative micrographs (a) and quantification (b) of colocalization between DsRed-Mito and GFP-Parkin (n = 11 per each group). Scale bar, 10 μm. Data are mean ± SEM; ****p < 0.0001 vs. control. c and d Immunoblotting analysis (c) and quantification (d) of the mitochondrial marker COX IV and LC3-II (n = 4 per each group). Data are mean ± SEM; COX IV; *p = 0.0291 vs. control, LC3-II; ****p < 0.0001 vs. control. [file 13041_2021_777_MOESM1_ESM.pdf]

Fig. S2

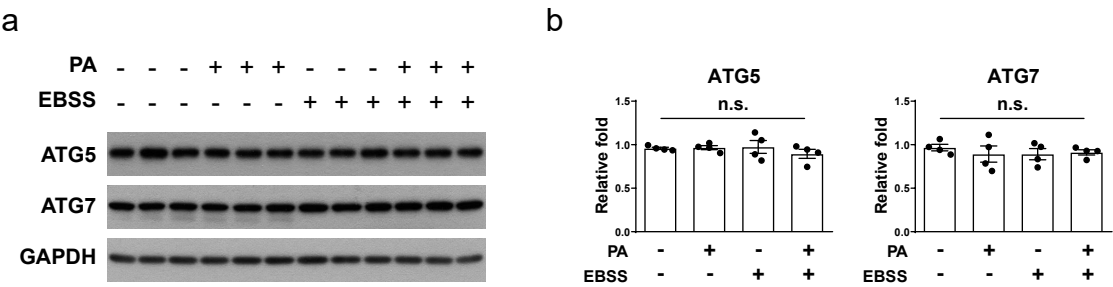

Supplement: Supplementary file 2 — Additional file 2: Fig. S2. Palmitate does not change the levels of ATG5 and ATG7. Cells were starved in EBSS for 3 h in the presence or absence of palmitate (0.1 mM). a and b Immunoblotting analysis (a) and quantification (b) of ATG5 and ATG7 (n = 4 per each group). Data are mean ± SEM; n.s., no significant difference. [file 13041_2021_777_MOESM2_ESM.pdf]

Fig. S3

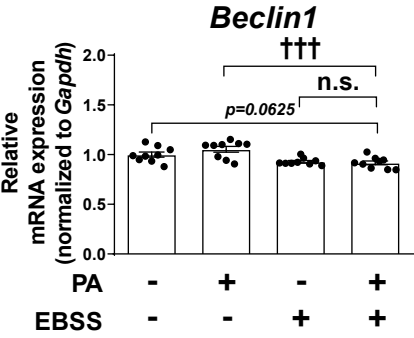

Supplement: Supplementary file 3 — Additional file 3: Fig. S3. The mRNA level of Beclin1 is not changed by palmitate and/or EBSS. Cells were starved in EBSS for 3 h in the presence or absence of palmitate (0.1 mM). a mRNA expression of Beclin1 (n = 9 per each group). Data are mean ± SEM; †††p = 0.0008. n.s., no significant difference. [file 13041_2021_777_MOESM3_ESM.pdf]

Fig. S4

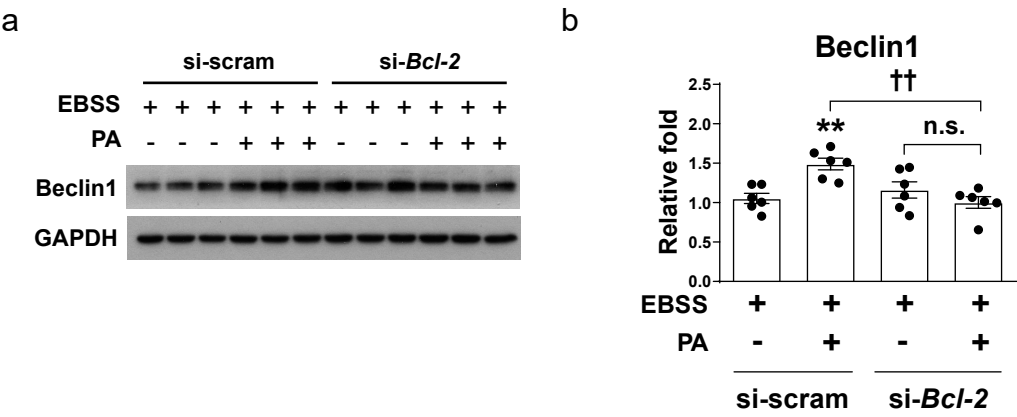

Supplement: Supplementary file 4 — Additional file 4: Fig. S4. Knockdown of Bcl-2 diminishes the increase of Beclin1 by palmitate under starvation. Cells transfected with si-scram or si-Bcl-2 were starved in EBSS for 3 h in the presence or absence of palmitate (0.1 mM). a and b Immunoblotting analysis (a) and quantification (b) of Beclin1 (n = 6 per each group). Data are mean ± SEM; **p = 0.0052 vs. EBSS in si-scram, ††p = 0.0019. n.s., no significant difference. [file 13041_2021_777_MOESM4_ESM.pdf]
